# Maxims nudge equitable or efficient choices in a Trade-Off Game

**DOI:** 10.1371/journal.pone.0235443

**Published:** 2020-06-30

**Authors:** Long Huang, Wansheng Lei, Fuming Xu, Hairong Liu, Liang Yu, Fujun Shi, Lei Wang

**Affiliations:** 1 School of Psychology, Jiangxi Normal University, Nanchang, People’s Republic of China; 2 School of Humanities and Management, Wannan Medical College, Wuhu, People’s Republic of China; 3 School of Educational Science, Nanning Normal University, Nanning, People’s Republic of China; Middlesex University, UNITED KINGDOM

## Abstract

The core problem in the distribution dilemma is the trade-off between equity and efficiency. With the development of socio-economic conditions, the optimal decision changes between equitable and efficient options. The methods for nudging decision-makers to make optimal decisions without changing the event are extremely important. This study used two laboratory behavior experiments to explore the impact of maxim information on the trade-off between equity and efficiency. The study explores whether stake levels and division schemes affect the nudging effect of the maxim in a Trade-Off Game (TOG). We found that participants were affected by maxim information in decision-making scenarios, and participants showed different equity preferences as the maxim information changed, without relevance of the stake level. Additionally, the nudging effect of the maxim only exists under the condition that the distributor's interests is not affected.

## Introduction

Efficiency and equity are important topics for human society, involving economic distribution, taxation, education, and pensions. The relationship between efficiency and equity can be controversial. While maintaining equity may come at the expense of total social welfare, the pursuit of maximizing social efficiency can lead to unfair results. This question of trade-off between efficiency and equity has been studied extensively in the fields of moral psychology and behavioral economics [1–4]. Utilitarian theories of distributive justice hold seek maximum benefits (happiness) or the efficiency sum, rather than equity, even if it results in no happiness [5]. Conversely, deontological theories of distributive justice argue that correct behavior (i.e., equity) is more important. Even if the distributive act maximizes the good but violates the principle advocated by equity, rights, or obligations, it is considered a moral error [6].

However, the equity and efficiency are not incompatible, with no absolute advantage or disadvantage. This is most obvious in economic development and distribution of benefits. American economist Okun believes that, "For efficiency, we must sacrifice some equity, and for equity, we must sacrifice some efficiency [7]." Equity and efficiency are alternate relations, and both must be considered. Kuznets' Inverted U-hypothesis implies that efficiency and equity are consistent in early stages of economic development [8]. With rapid economic growth, the contradiction between efficiency and equity increases; though at a certain high level, the two tend toward consistency again. It is necessary to balance equity and efficiency in economic distribution. Therefore, in the context of different times, places, and situations, distributive justice may not be constant and may be equity or efficiency. Some economically developed welfare countries in Europe and planned economic countries have implemented equity priority policies. Many developing countries have implemented efficiency priority policies in the early stages of economic development. Globally, most countries have implemented policies that priority to efficiency with due consideration to equity [9]. For example, with social and economic development, China has adjusted its income distribution policy from "distribution according to work" to "priority to efficiency with due consideration to equity." Now the policy is, "The relationship between efficiency and equity should be well handled in both primary distribution and redistribution." This adjustment responds to rapid economic development and the widening income gap. It is also very important to trade off between equity and efficiency in economic distribution policies at different levels, which are related to the well-being of all human beings. Up to this point, the importance of achieving efficiency often overrides concerns for equity in public health decision making at the national level. However, equity is more important than efficiency at the regional and local levels. Therefore, the optimal choice of decision-makers when trading off between equity and efficiency needs to be changed according to time, place, and situation, and it is particularly important to promote decision-makers to make optimal decision (equity or efficiency) based on actual needs [10].

How to promote decision-makers to make optimal decision when trading off between equity and efficiency? Thaler and Sunstein (2008) suggested that nudging can guide decision makers to make optimal choices. They believed that humanity’s limited reason and willpower lead to decision-making results that are often sub-optimal or inefficient. This irrational free choice needs correcting to help decision making [11]. Nudging is the use of cognitive biases (such as the framing effect, base rate fallacy, and heuristics) to influence decisions by changing the way information is expressed, rather than using obvious reward or punishment mechanisms or coercive means. As advocated by "libertarian paternalism", nudging should guide choices and behaviors as parents raise children, while allowing the right to choose freely without force. The goal is to help make right decisions. This nudge by non-monetary strategies has been applied to a range of behavioral interventions, including alcohol and drug use, eating disorders, gambling, recycling, and energy consumption, because it is inexpensive and easy to implement [12]. Thaler and Benartzi (2004) designed a default pension plan to increase individual savings in the United States [13]. Capraro and Rand (2018) first attempted to use nudging to promote equitable or efficient choices. They designed a "Trade-Off Game" (TOG) and found that participants preferred options described as "morally right" regardless of whether the option was equitable or efficient [14]. Subsequent researchers confirmed this conclusion, finding that the nudging effect mainly exists in the situations where stakes are low, and the participants' interests change greatly [15].

Similar nudges include maxims, slogans, and proverbs, which are often posted on the street, desk, or wall. The purpose of maxims is not only to transfer knowledge and information, but also to form an experience connected with the meaning they transfer. Their way of creating an experience is akin to creating a metaphorical meaning that depends on associations; thus providing the formation of images in the mind and creating an experience that one can compare with the situation one finds oneself in or a situation one might come across. maxims are one of the effective tools to guide daily life activities, especially with the help of their expressive power [16]. Two examples are: "Protect the environment, everyone has a responsibility;" and "Justice and equitable may be late but shall never absent." These maxims are aimed to draw attention to social norms, thereby affecting behavior. Research on social norms affecting behavior has found that individuals prefer to follow norms [14,17–22]. A natural experiment also found that social normative information is more effective than technical water conservation information in reducing household water consumption [23]. However, individuals do not always remember norms, and only when the individual focuses on a norm does it have an influence [24,25]. Recent psychological research shows that drawing attention to a norm is an important method to generate compliance behavior. In this "focused" state, individuals are more likely and/or faster to recall and associate words or behaviors related to these cues, which produce availability heuristics and influence behavior. Krupka and Weber (2009) found that focusing on the behavior of others in the environment can promote individual prosocial behavior [19]. Brewer and Gross (2005) use the information frame to focus participants' thoughts on a value, which affects overall thinking on the problem [26]. Obradovich and Guenther (2016) found that emphasizing collective responsibility increases economic support and energy-saving behavior for environmental protection [27]. Therefore, if the attention of decision-makers is directed to the information or behavior related to a certain social norm, decision-makers are more likely to follow this social norm and thus produce behavior that conforms to this social norm. This study aims to use maxims to change the environment, thereby forming a frame to influence behavior. This will have research significance and application value, as maxims are used frequently in daily life. While many people retain maxims, which may encourage or restrain, the question to study is: In what context and to what extent are maxims effective? As far as we know, few empirical studies have explored this issue.

## Study overview

This study explores the impact of maxims on decision making of equity and efficiency in the TOG. In the TOG, participants make a one-time, anonymous, unilateral choice about money distribution between themselves and two other players (without distribution rights). All three players earn 15 monetary units (MUs) in the equitable option (total amount: 45 MUs), or the three players earn different amounts (12 MUs, 15 MUs, 23 MUs), but the total is increased in the efficient option (total 50 MUs) [13,14]. The game design creates an equity and efficiency trade-off context. One scenario contains several persuasion maxims about equitability (e.g., delaying justice is injustice; the public and the peaceful are foundations of the country). The other scenario contains several persuasion maxims about efficiency (e.g., efficiency is the good work of the soul; there are only two kinds of material: high efficiency and low efficiency; there are only two kinds of people: high efficiency and low efficiency). If participants are not affected by maxims, regardless of the scenario, the distribute choice should be the same or similar, and there should be no significant difference in the equal preference of the scenarios. However, according to the nudging theory, availability heuristics, and empirical research [14,15,23], we speculate that participants’ equal preference is affected by relevant information, such as maxims and social norms. Therefore, we proposed the following hypothesis:

Hypothesis 1: Maxims have a nudging effect in the TOG, and it will affect the participant’s equal preference.

In Study 1, the efficient options were subdivided into three division schemes (12:15:23, 15:23:12, and 23:12:15, participant is first), corresponding to three situations of the participant’s reduced, unchanged, and increased interest. The stakes were set to 10 units in the TOG. This explores whether the nudging effect of maxims is affected by changes in the participant’s interests. Studies have divergent views on whether decision-making is affected by changes in the participant’s interests [14,15,28]. Therefore, we proposed the following hypothesis:

Hypothesis 2: The nudging effect of maxims is affected by changes in the participant’s interests.

Previous studies found that the stakes of the game had no effect on tasks [29,30]. However, recent studies found that the amount distributed to others will decrease in high-stake scenarios [31,32], especially in a unilateral game with no risk of loss [33]. Other studies found that in the trade-off between equity and efficiency, the proportion of decision-makers who choose equitable options and self-interest options increased in high-stake contexts, while the proportion of the options that have a large social benefit but reduced self-interest decreased [34]. Therefore, to explore the influence stake size on maxim nudging effect, Study 2 sets different stakes sizes: small (10, same as Study 1), medium (100), and high (1,000). We proposed the following hypothesis:

Hypothesis 3: The nudging effect of maxims is affected by the size of stakes.

## Study 1

### Materials and methods

#### Participants

We recruited 455 students (221 male/234 female; average age = 18.28 ± 0.84 years) from Wannan Medical College. Participants were told they would receive a 10 RMB remuneration after the study. An additional monetary reward depends on their income in the TOG, and the income is converted by a certain percentage as their additional reward. For example, if the participant gets 150 MUs in the task, the extra reward is 150 × 0.1 = 15 MUs. Therefore, the final reward for participants is 10 + 15 = 25 MUs. Each participant signed a written informed consent form. The monetary unit is RMB in our study, and exchange rate is 1 RMB = 0.141 USD. The study was approved by the Academic Ethics Committee of Wannan Medical College.

#### Design

The TOG used a 3x2 between-subjects design. The division schemes were 12:15:23, 15:23:12, and 23:12:15. The maxims were equity and efficiency. The options selected by participants represented the dependent variable.

Two rooms, with maxims of equity or efficiency on the walls, desks, and computer screensavers, were prepared. In the TOG, participants were randomly assigned to one of six situations. Under the efficiency condition, participants perform TOG on the computer in a room with efficient maxim. Under the equity condition, participants perform TOG on the computer in a room with equitable maxim. The stakes were set to 100 units in the TOG. The TOG is:

“You and players 2 and 3 are conducting an anonymous three-person economic distribution task. Player 2, player 3, and you are strangers. As the distributor, you can choose from the following two division schemes, but players 2 and 3 have no right to make decisions. Which option would you choose?

Option A: Each of you receives 150 MUs.
Option B: You receive 120 MUs (or 150 MUs, or 230 MUs), player 2 receives 150 MUs (or 230 MUs, or 120 MUs), and player 3 receives 230 MUs (or 120 MUs, or 150 MUs).”

#### Procedure

When the participants arrived at the lab, they were told they would receive basic rewards and game rewards for participating. Participants were then taken into rooms with maxims of equity or efficiency, and they were told that they would team up with two other anonymous players (players 2 and 3) online. In fact, the other two players were virtual computer players. After the team is completed, the participants perform the TOG on the computer.

### Results and discussion

Values were assigned to variables in statistics: maxim (0 = efficient, 1 = equitable) and division schemes (0 = 12:15:23, 1 = 15:23:12, 2 = 23:12:15), and participants’ choice as the dependent variable (0 = efficient, 1 = equitable). For example, if the average score of equal reference is 0.46 across the sample, it means that 46% of participants have equal preference. We fitted a binomial logistic regression model with two dummy variables as predictors (S1 Table).

**Fig 1:**
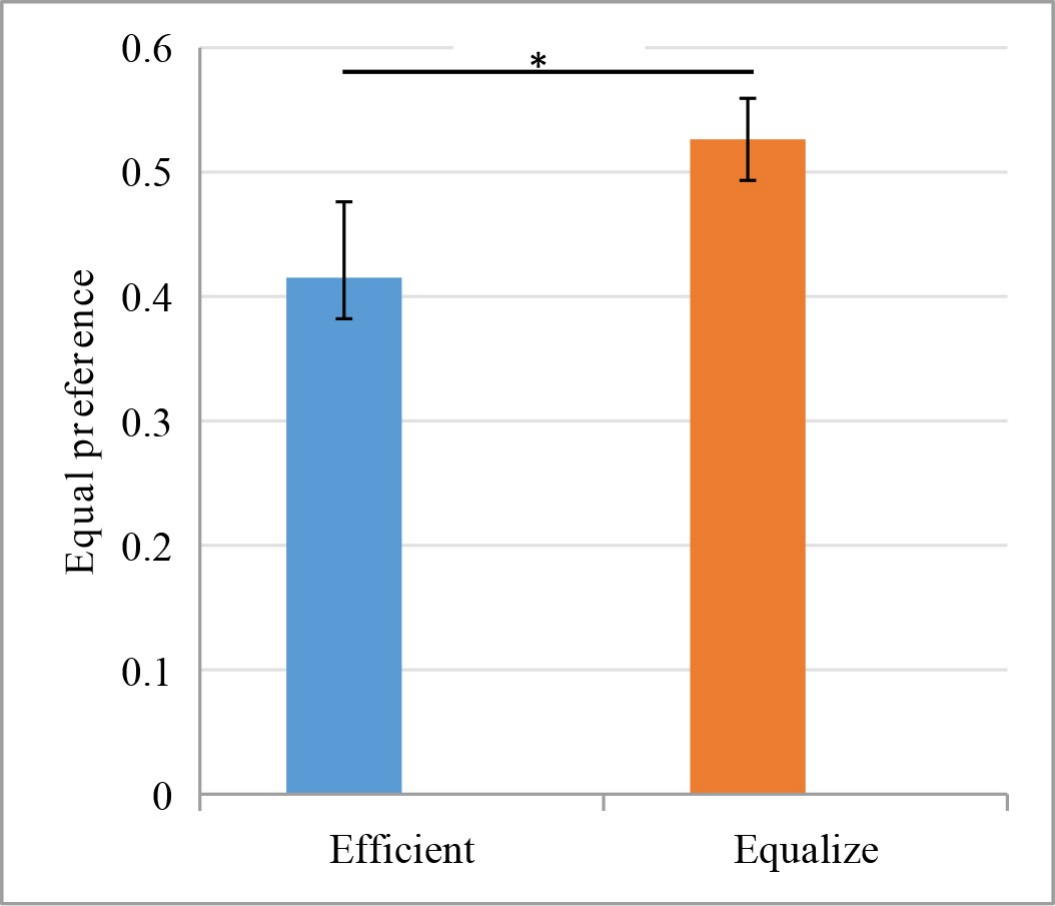
Equity preference under different maxims conditions.

Consistent with Hypothesis 1, maxim messages affected TOG choice as expected: Odds Ratio (OR) = 1.61, 95% CI [1.10, 2.37], p = 0.014 (Fig 1). Most participants chose the equitable option under equitable maxims condition (52.63%), whereas fewer participants chose equitable under the efficient maxim condition (41.51%).

**Fig 2:**
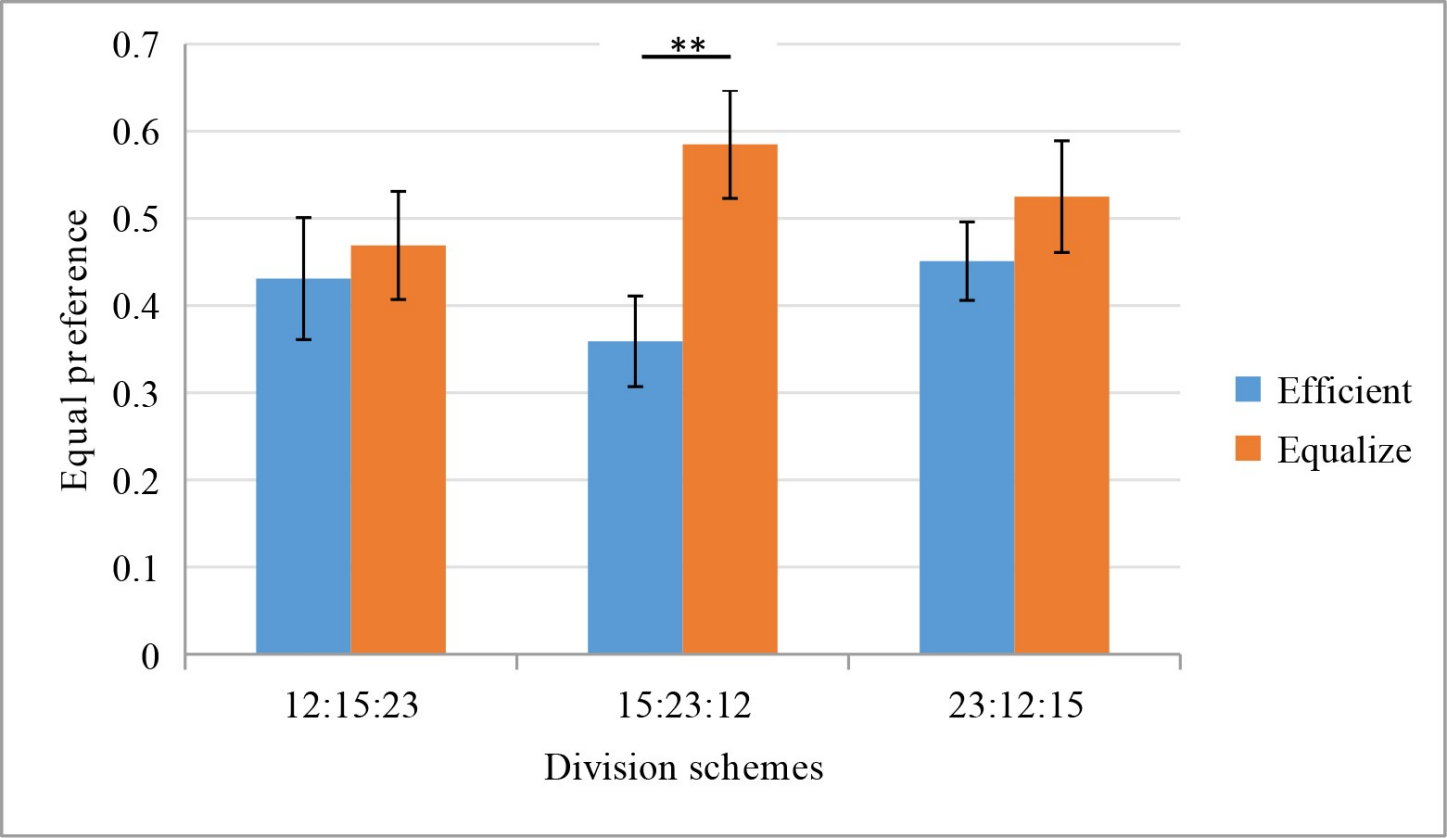
Equity preference under different maxims and division schemes conditions.

Consistent with Hypothesis 2, there is a statistically significant interaction between maxim messages and division schemes: OR = 0.31, 95% CI [1.01, 1.70], p = 0.04. The nudging effect of maxims is influenced by the division schemes in the efficient option. Under the 15:23:12 division scheme, participants chose the equitable option under the equitable maxims condition (58.50%) significantly more than participants who chose this option under the efficient maxims condition (35.90%): χ^2^_(1)_ = 7.85, p = 0.005. φ = 0.22. Under the division schemes of 12:15:23 (χ^2^_(1)_ = 0.16, p = 0.69) and 23:12:15 (χ^2^_(1)_ = 0.89, p = 0.35), there is not a statistically significant difference between participants choosing the equitable option under the equitable maxims condition and the efficient maxims condition (Fig 2). This indicates that the nudging using maxims is effective only in cases where the distributor's interests are not affected, which may indicate that the distributor's interests impact decision-making. When stakes are high, the distributor’s interests will also change. In Study 2, three game stakes (small, medium, and high) are set to examine the impact of the stakes on the nudging effect of maxims.

## Study 2

### Materials and methods

#### Participants

We recruited 567 students (280 male/287 female; average age = 18.26 ± 1.15 years) from Wannan Medical College. Same as Study 1, participants would receive a basic remuneration of 10 MUs and additional money as a percentage of one choice at random in the three TOGs. Study 2 participants did not participate in Study 1, and all signed written informed consent forms. The study was approved by the Academic Ethics Committee of Wannan Medical College.

#### Design

A mixed design of 3 (division schemes: 12:15:23, 15:23:12, 23:12:15) x 2 (maxims: equity, efficiency) x 2 (stakes: small, medium, high) was used. The stakes are within-subjects variable, division schemes and maxims are between-subjects variables, and the options selected by participants are the dependent variables in the TOG. The TOG used in Study 2 is the same as Study 1. However, we set three different stakes: small (unit: 10, same as Study 1), medium (unit: 100), and high (unit: 1,000). Participants completed three TOGs, one for each stake level. The order was balanced among the subjects (S1 File).

#### Procedure

Same as study 1.

### Results and discussion

A repeated measure ANOVA was performed on equal preference with stakes (0 = small, 1 = medium, 2 = high) for the within-subject factor, with maxims (0 = efficient, 1 = equitable), and with division schemes (0 = 12:15:23, 1 = 15:23:12, 2 = 23:12:15) for the between-subject factors. The result of Mauchly’s test of sphericity as a measurement of the main effect w significant (p < .05), which means that the three factors did not meet the sphericity assumption, so we used the results after the Greenhouse–Geisser correction.

**Fig 3:**
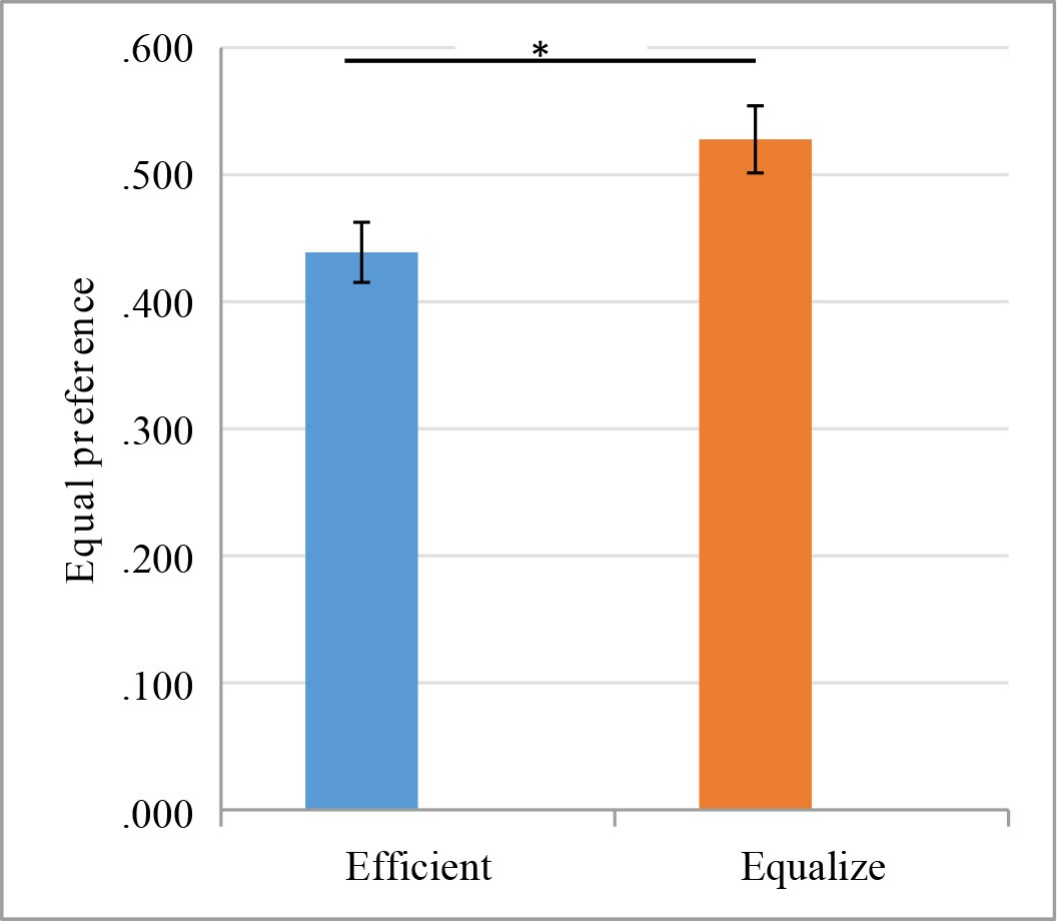
Equity preference under different maxims conditions.

Consistent with Hypothesis 1 and Study 1, the results of Study 2 indicate that the effect of maxims is significant: F(1,561) = 6.27, p = 0.013, η_p_^2^ = 0.011 (Fig 3). Most participants chose the equitable option under the equitable maxims condition (52.78%), where fewer participants chose the equitable option under the efficient maxims condition (43.89%).

**Fig 4:**
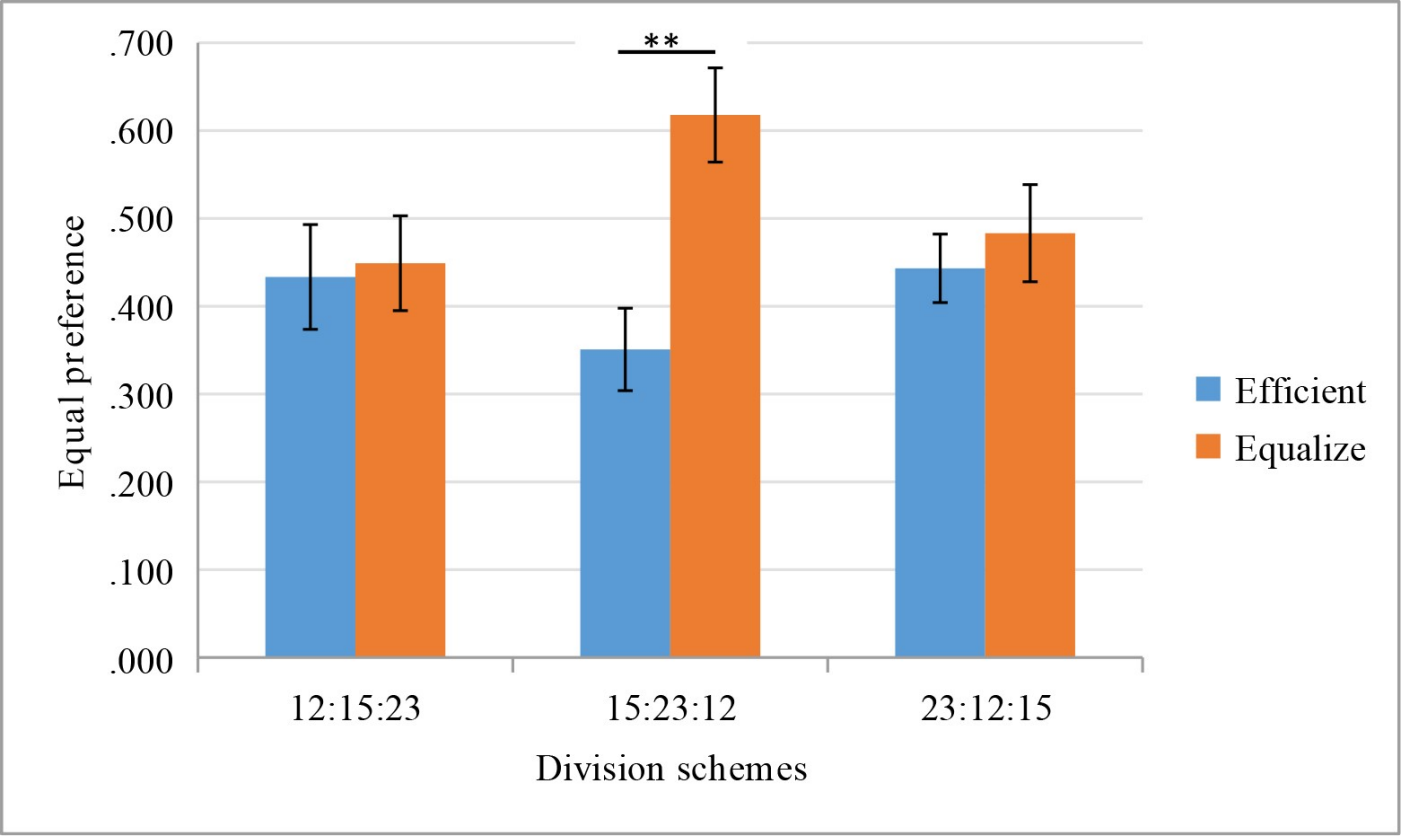
Equity preference under different maxims and division schemes conditions.

Consistent with Hypothesis 2 and Study 1, there is a statistically significant interaction between maxims and division schemes: F(2,561) = 3.55, p = 0.029, η_p_^2^ = 0.013. The nudging effect of maxims is influenced by the division schemes in the efficient option. Simple effects analysis found that under the division scheme of 15:23:12, participants chose the equitable option under the equitable maxims condition (61.78%) significantly more than participants who chose this option under the efficient maxims condition (35.08%), p = 0.0002. Under the division scheme of 12:15:23 (p = 0.86) and 23:12:15 (p = 0.28), there is not a statistically significant difference between participants choosing the equitable option under the equitable maxims condition and the efficient maxims condition (Fig 4).

**Fig 5:**
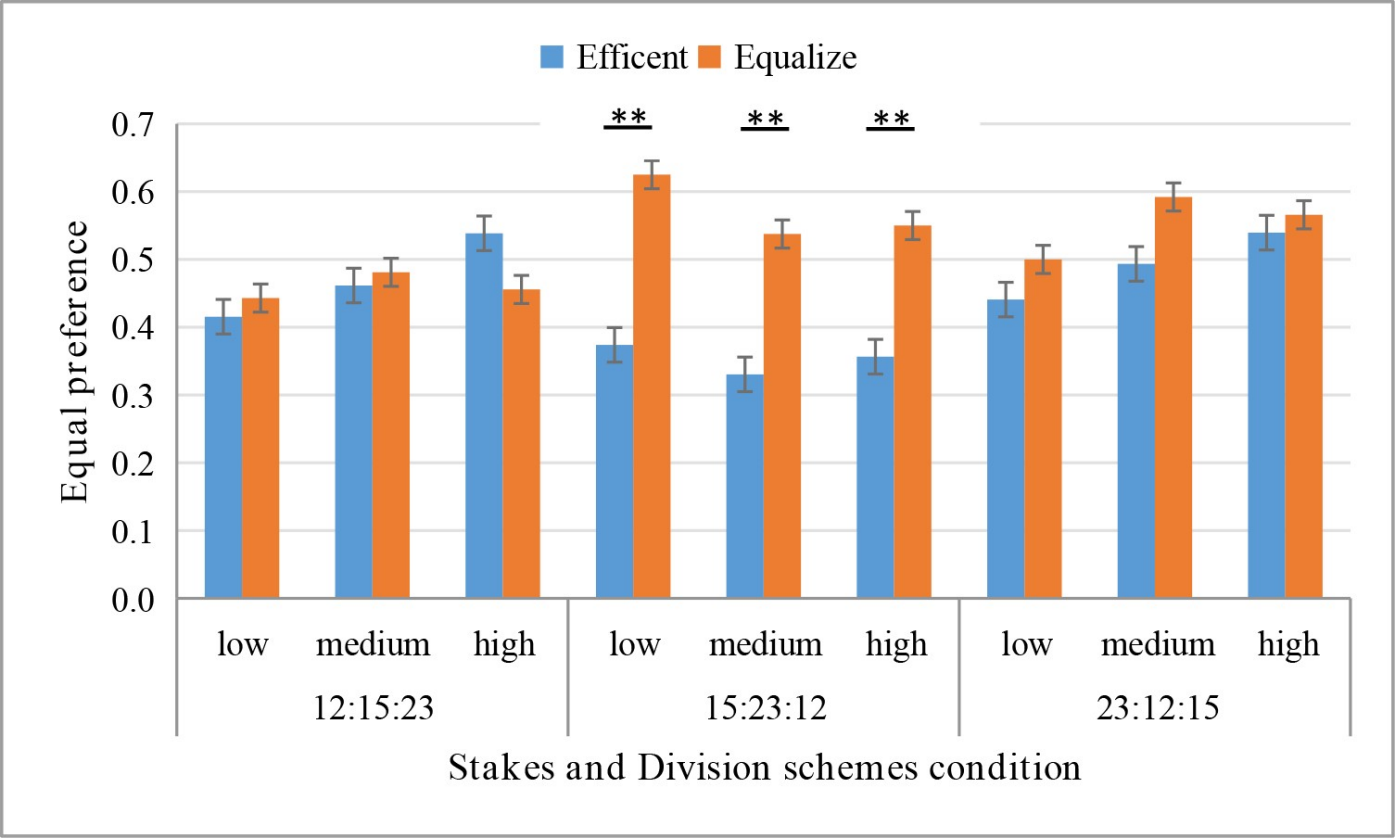
Equity preference under different stakes, maxims, and division schemes conditions.

Inconsistent with Hypothesis 3, the effect of stakes is not significant (p = 0.28). The stakes have no effect on the nudging effect of maxims. Chi-square test analysis found that under the division scheme of 15:23:12, regardless of whether the stake is small (χ^2^ (1) = 11.92, p = 0.001. φ = 0.25), medium (χ^2^ (1) = 8.33, p = 0.004. φ = 0.21), or high (χ^2^ (1) = 7.18, p = 0.007. φ = 0.19), participants chose the equitable option under the equitable maxims condition significantly more than participants who chose this option under the efficient maxims condition. Under the division schemes of 12:15:23 and 23:12:15, regardless of whether the stake is small, medium, or high, participants chose the equitable option with no statistically significant difference between the equitable maxims condition and the efficient maxims condition (all ps > 0.1) (Fig 5).

## General discussion

The core problem in the distribution dilemma the trade-off between equity and efficiency, both of which are supported by research theories [5,6] and are considered types of prosocial behavior. With the development of socio-economic conditions, the optimal decision between equitable and efficient options change. Guiding decision makers to make optimal decisions according to needs is an important issue. This study demonstrates that maxim information in the environment will nudge decision preferences, especially in the situation where the distributor's interest does not change. Additionally, this result is not affected by the stakes.

Previous studies found that small psychological changes can influence decision-making behaviors with real consequences [35]. In the decision-making scenario, small changes in the wording of information may induce psychological changes [36,37] and affect decision making. The results of Studies 1 and 2 confirm this conclusion, which demonstrates that participants are affected by maxim messages in the decision-making environment. When maxims convey relevant information about equity, more participants choose equitable options and show equitable preferences. When maxims convey relevant information about efficiency, more participants choose efficient option and show efficient preferences. This result is consistent with similar studies. Brañas-Garza (2007) added a “non-neutral” sentence at the end of the dictator game: “Note that your recipient relies on you.” The frame group with “non-neutral” sentences, compared to the baseline group without “non-neutral” sentences, was shown to have more altruistic behavior and allocated more money to others [17]. Similarly, context-induced empathy (requiring someone to “put themselves in the shoes of others”) leads to increased prosocial behavior [38,39]. Attaching relevant pictures of “Your taxes finance important public services” can increase the amount of overseas income reported by people when applying for tax payment [40].

The results of Studies 1 and 2 show that the nudging effect of maxims only exists under the condition that the efficient option is 15:23:12. Compared with the equitable option, the participant's own interests is the same regardless of whether the equitable or efficient option is selected. This indicates that the nudging effect of the environmental framework (maxim) only exists in the case where the distributor's interests are not affected, which may indicate that the distributor's interests impact decision-making. Research indicates that topics involving high levels of personal input are less susceptible to attribute frame effects. Marteau (1989) did not find a frame effect on various issues involving abortion decisions [41]. The degree of relevance of the participants determines the psychological distance to the event. The psychological distance impacts different levels of understanding, which affects cognition, preference, and behavior. The greater distance from the direct experience, the more abstract the interpretation or consideration [42]. In this study, when the participants' interests remain unchanged, the psychological distance is far, and the environmental frame effect is obvious. Conversely, when the participants' interests change, psychological distance is close, and the environmental frame effect is not obvious. This is consistent with results on psychological distance and frame effects in China [43]. McElroy and Mascari (2007) found that the frame effect is not obvious when psychological distance is close, while the frame effect is significant when psychological distance is far [44]. There are few studies on change in the distributor's interests on the nudging effect, and conclusions are inconsistent. Capraro and Rand (2018) found that the moral frame can significantly influence the distributor's equal and efficient preferences [14]. Huang et al. (2019) found that when participants' interest changes greatly, the effect of the moral frame is more significant [15]. However, these two studies require participant responsibility for the decision whether to change the existing division scheme, which may explain the difference those results and the results of our study. A study similar to this study,which participants are free to choose between (13,13,13) and (13,23,13), indicates that when the distributor's amount does not change, the moral frame effect is significant [28], which is a consistent result with our study. However, the study does not further examine when the distributor's interests change. Additionally, maxim information and moral information are different, and maxim information may not impose moral costs on participants when making decisions.

Under the conditions where participants’ interest changes (12:15:23 and 23:12:15), the difference in the proportion of participants’ equitable choices under the two maxim frame is not significant, both between 0.4–0.5. The result shows that participants are not influenced by the maxims frame, and regardless if their interests are impaired or benefited, there is a tendency to choose efficiency options, although the difference is not significant. This may have to do with the Chinese background of subjects in our study. China's income distribution policy was "give priority to efficiency with due consideration to fairness," emphasizing social efficiency. While participants have a general efficiency preference, under the conditions of 23:12:15, the efficiency preference is not obvious. This aligns with participant economic interests and does not meet the efficiency-first allocation policy. This result may have two explanations, though we lack definitive evidence for either. First, participants in the study (Chinese college students) were influenced by Confucian culture from an early age, and their main cultural orientation is collectivism, with a strong sense of group responsibility and pursuit of harmony and conformity [45]. Collectivism emphasizes group consciousness, harmony, emotional interdependence, obligations, and group solidarity [46,47]. Participants in our study may be collectivists and reluctant to show selfishness in benefits distribution, nor are they willing to bear undermining group consistency. Therefore, they are more willing to forego high-value benefits to maintain group consistency. Second, this result is also consistent with the fairness theory of Adams (1965), where distributors receiving too much will reduce people's sense of fairness [48]. Even when people can obtain more, they tend to relinquish their interests and maintain fairness [49]. Additionally, studies have found that Chinese college students generally have high empathy [50]. Individuals with high empathy may respond negatively to messages that reframe prosocial acts as consistent with self-interest, preferring to view such acts as egoistic [51]. This may result in participants not preferring efficiency options when their interests are consistent with the maxim information.

Finally, Study 2 found that whether the stakes are small, medium, or high in the TOG, participant behavior is affected slightly. Specifically, when the efficient division scheme is 15:23:12, at any stake level, more participants choose the equitable option when the maxims are equitable information than when the maxims are efficient information. In the other two division schemes, at any stake level, participants' equity preference when the maxims are equitable information show no difference from the equity preference when the maxims are efficient information. This shows that participants are more concerned if their interests will change compared to other options, rather than the stakes. When compared with other options, where participant interests will not change and at any stake level, the equity preference of participants will be affected by the nudging effect of maxims in the environment. However, the compensation given to participants is converted according to a percentage of the game interest, and participants' true psychological cognitive values of the interests may be smaller. Therefore, our conclusion may need further research in the natural environment to verify and improve the effectiveness of the results. Additionally, the amount of small stakes is 150 RMB (21 USD) in our Study 2, which may still be a bit big stakes. Therefore, we need to further explore the situation at smaller stakes level in the future. Finally, participants in this study were medical students, and more caution is necessary in generalizing the findings for a more general population.

## Supporting information

S1 Table Binomial logistic regression of participants' equal preference in study 1. (DOCX)

S1 File The TOG in the study 2. (DOCX)
