# Supplementary material for: Maxims nudge equitable or efficient choices in a Trade-Off Game
Source: PLoS One. 2020 Jun 30;15(6):e0235443. doi: 10.1371/journal.pone.0235443 (PMC7326161; doi:10.1371/journal.pone.0235443)
Supplement: S1 Table — (DOCX) [file pone.0235443.s001.docx]

S1 Table. Binomial logistic regression of participants' equal preference in Study 1

| Variables | B | SE | Wals | OR | *p* | CI |
| --- | --- | --- | --- | --- | --- | --- |
| maxim | 0.479 | 0.195 | 6.023 | 1.614 | 0.014 | 1.10-2.37 |
| division scheme | 0.104 | 0.121 | 0.736 | 1.11 | 0.391 | 0.88-1.41 |
| maxim×division scheme | 0.272 | 0.132 | 4.223 | 1.313 | 0.04 | 1.01-1.70 |
