# Supplementary material for: Maxims nudge equitable or efficient choices in a Trade-Off Game
Source: PLoS One. 2020 Jun 30;15(6):e0235443. doi: 10.1371/journal.pone.0235443 (PMC7326161; doi:10.1371/journal.pone.0235443)
Supplement: S1 File — (DOCX) [file pone.0235443.s002.docx]

**S1 File. The TOG in the study 2**

**Small stakes (unit: 100, same as study 1) in the TOG:**

“You and players 2 and 3 are conducting an anonymous three-person economic distribution task. Player 2, player 3, and you are strangers. As the distributor, you can choose from the following two division schemes, but players 2 and 3 have no right to make decisions. Which option would you choose?

Option A: Each of you receives 150 MUs.

Option B: You receive 120 MUs (or 150 MUs, or 230 MUs), player 2 receives 150 MUs (or 230 MUs, or 120 MUs), and player 3 receives 230 MUs (or 120 MUs, or 150 MUs). ”

**Medium stakes (unit: 1000) in the TOG**

“You and players 2 and 3 are conducting an anonymous three-person economic distribution task. Player 2, player 3, and you are strangers. As the distributor, you can choose from the following two division schemes, but players 2 and 3 have no right to make decisions. Which option would you choose?

Option A: Each of you receives 1500 MUs.

Option B: You receive 1200 MUs (or 1500 MUs, or 2300 MUs), player 2 receives 1500 MUs (or 2300 MUs, or 1200 MUs), and player 3 receives 2300 MUs (or 1200 MUs, or 1500 MUs). ”

**High stakes (unit: 10000) in the TOG**

“You and players 2 and 3 are conducting an anonymous three-person economic distribution task. Player 2, player 3, and you are strangers. As the distributor, you can choose from the following two division schemes, but players 2 and 3 have no right to make decisions. Which option would you choose?

Option A: Each of you receives 15000 MUs.

Option B: You receive 12000 MUs (or 15000 MUs, or 23000 MUs), player 2 receives 15000 MUs (or 23000 MUs, or 12000 MUs), and player 3 receives 23000 MUs (or 12000 MUs, or 15000 MUs). ”
